# Rice Husk-Derived MCM-41 for Efficient Hg(II) Removal: Performance, Mechanism, and Environmental Safety in Real Water Matrices

**DOI:** 10.3390/nano16110694

**Published:** 2026-06-01

**Authors:** Naren Bocanegra, Marcela Paredes-Laverde, Nancy Acelas, Ximena Carolina Pulido, Luis Rodríguez, César Jaramillo-Páez

**Affiliations:** 1 Grupo de Investigación en Química Aplicada a Procesos Ecológicos (QUAPE-UT), Facultad de Ciencias, Universidad del Tolima, Ibagué 730006, Colombia; nbocanegram@ut.edu.co (N.B.); xpulido@ut.edu.co (X.C.P.); lfrodriguezh@ut.edu.co (L.R.); 2 Grupo de Investigación Navarra Medicina, Facultad de Ciencias de la Salud, Fundación Universitaria Navarra-Uninavarra, Neiva 410010, Colombia; marcela.paredes@udea.edu.co; 3 Grupo de Investigación Materiales con Impacto (Mat&mpac), Facultad de Ciencias Básicas, Universidad de Medellín, Medellín 050026, Colombia

**Keywords:** heavy metal, mesoporous material, agro-industrial waste, adsorption, ecosystem protection

## Abstract

Mercury contamination in water poses severe environmental and health risks, requiring efficient and sustainable removal strategies. In this study, rice husk (RH), rice husk-derived materials, including rice ash (RHA), and Mobil Composition of Matter No. 41 (MCM-41) were evaluated as adsorbents for Hg(II) removal in aqueous systems. Among the tested materials, MCM-41 exhibited superior adsorption performance, achieving up to 98% Hg(II) removal under optimal conditions (pH 6.8, 3 g L^−1^ of adsorbent, and a pollutant concentration of 0.90 mg L^−1^). Adsorption followed a pseudo-second-order kinetic model and was best described by the Langmuir isotherm, indicating monolayer adsorption. The maximum adsorption capacity reached 0.80 mg g^−1^. Thermodynamic analysis revealed that the process was spontaneous and exothermic, primarily governed by coordination interactions and hydrogen bonding with surface silanol groups. The adsorbent’s applicability was further assessed in distilled water, synthetic industrial wastewater, and river water. Although high removal efficiencies were maintained, a decrease was observed in complex matrices due to competition from coexisting ions. Reusability tests demonstrated that MCM-41 retained its performance over four adsorption cycles. Environmental safety was evaluated through ecotoxicological and microbiological assays. *Daphnia magna* exhibited high sensitivity to Hg(II) (EC_50_ values of 0.0220 mg L^−1^ at 24 h and 0.0158 mg L^−1^ at 48 h), while treated samples showed improved germination indices of *Lactuca sativa*, particularly in distilled and river water. However, residual toxicity persisted in industrial wastewater matrices. Overall, rice husk-derived MCM-41 is a promising and sustainable adsorbent for Hg(II) removal, though further optimization is needed to mitigate residual toxicity in complex water matrices.

## 1. Introduction

Mercury (Hg(II)) is a highly toxic and persistent heavy metal that poses significant risks to aquatic ecosystems and human health due to its bioaccumulation and long-term environmental persistence. It can enter water bodies through various anthropogenic activities, including mining and industrial discharges, where it remains dissolved and difficult to remove [1]. Notably, the presence of Hg(II) has been reported in rivers and industrial waters at concentrations of 0.0006–0.0023 mg L^−1^ [2] and 0.7–3.8 µg L^−1^ [3], respectively. Therefore, the development of efficient and sustainable strategies for Hg(II) removal from water is critical.

Among the available treatment technologies, adsorption has emerged as one of the most effective methods due to its operational simplicity, high efficiency, and versatility. In this context, the use of low-cost adsorbents derived from agro-industrial residues has gained increasing attention as a sustainable alternative. Rice husk (RH), an abundant agricultural by-product representing approximately 20% of rice grain weight, is generated in quantities exceeding 150 million tons annually worldwide, creating both an environmental challenge and opportunity for valorization [4,5].

In its natural form, RH has demonstrated potential as an adsorbent due to its functional groups, which can bind metal ions. Furthermore, thermal treatment produces rice husk ash (RHA), a silica-rich material containing up to 95% silica [6], which can be further utilized as a precursor for synthesizing of advanced adsorbents. In particular, mesoporous materials such as MCM-41, characterized by high surface area, uniform pore structure, and abundant silanol groups, have shown excellent performance in heavy-metal adsorption. However, their high production cost and limited reusability remain important limitations.

Although rice husk-derived materials have been investigated for Hg(II) removal, most studies have focused on individual materials evaluated under simplified or idealized conditions, making cross-material comparisons unreliable. A systematic evaluation of natural RH, RHA, and MCM-41 derived from the same precursor remains scarce. This gap hinders a mechanistic understanding of how progressive physicochemical transformations in texture, morphology, surface chemistry, and pore structure govern adsorption performance. Consequently, the structure–performance relationships along the transformation pathway from raw biomass to engineered mesoporous silica remain insufficiently established, limiting the rational design of efficient and sustainable adsorbents for Hg(II) removal. Beyond material-level comparisons, performance in complex water matrices, such as industrial wastewater and river water, remains insufficiently explored. Furthermore, the environmental safety of treated water, particularly with respect to ecotoxicity and phytotoxicity, is rarely assessed, leaving a critical gap between technical adsorption performance and real-world water quality outcomes.

Therefore, this study aims to valorize rice husk as a precursor to produce three types of adsorbents (RH, RHA, and MCM-41), and to evaluate their performance for Hg(II) removal from aqueous systems. Special emphasis is placed on comparing their adsorption efficiency, elucidating the removal mechanism, assessing reusability, and evaluating their applicability in real water matrices. Additionally, phytotoxicity, ecotoxicity, and microbiological analyses were performed to assess the environmental safety of the treated water.

## 2. Materials and Methods

### 2.1. Reagents

Sodium hydroxide and cetyltrimethylammonium bromide (CTAB) were obtained from Panreac, while hydrochloric acid (37%) was purchased from Honeywell. Hg(II) was obtained from HgCl_2_ (Panreac). Other chemical reagents, including sodium bicarbonate (NaHCO_3_), calcium chloride dihydrate (CaCl_2_·2H_2_O), magnesium sulfate heptahydrate (MgSO_4_·7H_2_O), and potassium chloride (KCl), obtained from Microbiotests, were used to prepare a saline solution (Supplementary Material (SM) Table S1) for ecotoxicity testing. The synthetic industrial wastewater was prepared in the laboratory according to previously reported [7,8] (Table S2), and the chemical components were supplied by Panreac and Alpha Chemika. The real sample of river water was collected from the Cuenca Alta, Pradera, and Curillo (Caquetá, Colombia). Romaine lettuce used in the assays was provided by Ansac, and glyphosate was supplied by Monsanto. Daphnia magna was acquired from Microbiotests, while Chromocult tests were obtained from Merck, Germany.

### 2.2. Preparation of Adsorbents

Rice husk was obtained from agro-industrial sources in Espinal (Tolima, Colombia), thoroughly washed with distilled water, and subjected to acid leaching using 37% HCl (15 mL g^−1^ of the raw material) under stirring (240 rpm) for 5 h. The resulting material was dried and labeled as RH. A portion of the RH was calcined at 600 °C (10 °C min^−1^, 5 h) under an oxygen atmosphere to obtain rice husk ash (RHA). Subsequently, 15 g of RHA was refluxed in 2 M NaOH at 80 °C for 15 h. After cooling, the mixture was filtered, yielding sodium silicate. The extracted sodium silicate was subsequently used as the silica source for the synthesis of MCM-41.

MCM-41 was synthesized using CTAB as a template. Briefly, 1 g of CTAB was dissolved in 50 mL of distilled water under vigorous stirring at room temperature for 2 h. Subsequently, 10 mL of sodium silicate extracted from RHA was added, and the pH was adjusted to 10. The resulting suspension was stirred at 500 rpm for 15 h and then subjected to hydrothermal treatment at 100 °C for 24 h. The synthesized solid was recovered by vacuum filtration, thoroughly washed with ultrapure water, and dried at 60 °C for 24 h. Finally, the material was calcined at 550 °C for 6 h under an oxygen atmosphere to remove the template and generate the mesoporous structure, resulting in the final product, denoted as MCM-41.

### 2.3. Characterization Techniques

The adsorbents were characterized by X-ray diffraction (XRD) using a MiniFlex 600 diffractometer with Cu Kα radiation. Interplanar spacing (*d*_hkl_) was calculated using Bragg’s equation [9] (Text S1). Phase identification was performed using reference patterns for cellulose (ICDD card No. 00-060-1502) [10] and amorphous silica (ICDD card No. 00-001-0424) [11], and the PDF-4 Minerals 2025 database for mesoporous silica MCM-41 (ICDD card No. 00-074-1331). Elemental composition was determined by X-ray fluorescence (XRF) using a Nex QC+ system. Morphology and surface composition were analyzed by scanning electron microscopy (SEM) coupled with energy-dispersive X-ray spectroscopy (EDS) on a Thermo Fisher Scientific Scios 2 LoVac instrument. Thermogravimetric analysis (TGA) was performed using a TA Instruments SDT 650 analyzer.

Nitrogen adsorption–desorption isotherms were obtained using a 3Flex Micromeritics instrument. The specific surface area was calculated using the Brunauer–Emmett–Teller (BET) method, while pore size distribution was determined from the desorption branch using the Barrett–Joyner–Halenda (BJH) model. Total pore volume was calculated at a relative pressure P/Po = 0.993. Fourier-transform infrared (FT-IR) spectra were recorded in the range of 4000–400 cm^−1^ using a Spectrum Two spectrometer. The point of zero charge (pH_PZC_) was determined by the solid addition method [12].

Surface chemical states were analyzed by X-ray photoelectron spectroscopy (XPS) using the A. Centeno XPS/ISS/UPS platform, equipped with a 2-CMOS 150 analyzer and an Al Kα source (FOCUS 500, 100 W). Spectra were recorded with pass energies of 100 eV for survey scans and 20 eV for high-resolution measurements, employing charge compensation with a Flood Gun (FG 15/40-PS FG 500) operated at 94 µA and −3.6 eV.

### 2.4. Adsorption of Hg(II) with Analysis of Kinetic and Isotherm Models

Batch adsorption experiments were conducted by contacting 0.5 g L^−1^ of RH, RHA, and MCM-41 adsorbents with 50 mL of Hg(II) solution (0.90 mg L^−1^) at pH 6.8, 270 rpm, and 25 °C for 1440 min. Samples were collected at predetermined time intervals until equilibrium was reached. The percentage of Hg(II) adsorption was then calculated for each material (Text S2). All batch adsorption experiments were conducted in duplicate, and the results are reported as mean values. Good agreement between replicates and low standard deviations were observed and, where appropriate, are presented in graphical or tabular form. Also, given the good reproducibility observed between duplicate experiments, no additional statistical analyses were performed.

Adsorption kinetics of Hg(II) onto RH, RHA, and MCM-41 were analyzed using the pseudo-first-order and pseudo-second-order nonlinear models (Texts S3 and S4, respectively). The best-performing adsorbent was selected based on removal efficiency, the correlation coefficient (R^2^), and the average percentage error (APE, %) (Text S5). This systematic screening strategy was deliberately designed to focus subsequent efforts on the most promising candidate, ensuring a more robust and meaningful evaluation under real water matrix conditions and environmental safety assessments.

The selected material was further evaluated under varying conditions of adsorbent dosage (0.5–10 g L^−1^), pH (2–11) and initial Hg(II) concentration (0.64–18.0 mg L^−1^). Equilibrium data were fitted to the Langmuir and the Freundlich isotherm nonlinear models (Text S6 and Text S7, respectively). Model comparison criteria for the adsorption experiments were based on the coefficient of determination (R^2^) and the absolute percentage error (APE %). The model with the highest R^2^ values and lowest APE (%) was considered to select the model that best described the adsorption process. All kinetic and isotherm analyses were performed using Origin 2019b (32-bit) software.

### 2.5. Reuse Experiments

After the adsorption process, the spent adsorbent was dried at room temperature and regenerated by treatment with 0.1 M HCl until complete desorption of Hg(II) was achieved. The material was then filtered, thoroughly washed with distilled water, and dried at 100 °C for 24 h. The regenerated MCM-41 was reused in successive adsorption–desorption cycles following the same procedure. After four cycles, changes in the structural and textural properties of the material were evaluated using XRD, SEM, nitrogen physisorption, and FTIR analyses.

### 2.6. Thermodynamic Analysis and Proposed Adsorption Mechanism

Thermodynamic parameters for Hg(II) adsorption were determined at three temperatures (25, 45, and 65 °C) under fixed experimental conditions (initial Hg(II) concentration of 0.90 mg L^−1^, adsorbent dosage of 3.0 g L^−1^, pH 6.8, and stirring at 270 rpm for 12 h in a hermetically sealed system to prevent evaporation losses). Standard Gibbs free energy (ΔG°), enthalpy (ΔH°), and entropy (ΔS°) changes were calculated as described in Text S8. The adsorption mechanism was proposed based on a combined analysis of thermodynamic, kinetic, and isotherm results, supported by FTIR characterization of MCM-41 before and after Hg(II) adsorption.

### 2.7. Applications in Aqueous Matrices with Ecotoxicological and Microbiological Tests

The performance of MCM-41 for Hg(II) removal was evaluated in distilled water and compared with more complex aqueous matrices, including synthetic industrial wastewater and real river water. Adsorption experiments were conducted at an initial Hg(II) concentration of 0.90 mg L^−1^, adsorbent dose of 3 g L^−1^, natural pH (6.8–7.0), and a stirring rate of 270 rpm for 24 h.

The ecotoxicity of treated samples was assessed using acute toxicity tests with *Daphnia magna*, following OECD guideline 202 and ISO 6341 [13]. Neonates (<24 h old), obtained from ephippia hatched under controlled conditions (22 °C, continuous illumination), were exposed to treated samples for four days. Newly hatched neonates (<24 h old) were fed with the microalga *Spirulina* three hours before exposure to ensure optimal physiological condition.

Assays were performed in multi-well plates containing 10 mL of a saline solution enriched with water obtained after the process of Hg(II) adsorption using MCM-41 in distilled water, river water, and industrial wastewater. Five neonates were introduced into each well. Potassium dichromate K_2_Cr_2_O_7_ and distilled water were used as positive and negative controls, respectively. Plates were sealed with parafilm, covered with their respective lids, and incubated in the dark at 22 °C for 24 h. Subsequently, the percentage of *Daphnia magna* immobilization was reported. All experiments were conducted in quadruplicate.

The median effective concentration (EC_50_) of Hg(II) on *Daphnia magna* was determined under identical conditions at concentrations ranging from 0.0031 to 0.075 mg L^−1^, with exposure times of 24 and 48 h. EC_50_ values were calculated by Probit analysis using freely available statistical software.

Microbiological analysis was performed by filtering 100 mL of each sample (before and after treatment) through 0.45 µm membranes. The resulting membranes were placed in Petri dishes on nutrient pads impregnated with Chromocult coliform medium, prepared according to the manufacturer’s instructions [14] and pre-moistened with 3.0–3.5 mL of sterile water. All procedures were performed in triplicate, and the plates were subsequently incubated in the dark at 37 °C for 48 h. After incubation, total coliforms were identified as pink to red colonies, whereas *Escherichia coli* was identified by dark blue to violet colonies, based on the medium’s chromogenic response.

### 2.8. Phytotoxicity Assays

Phytotoxicity was evaluated using a seed germination assay with romaine lettuce (*Lactuca sativa*). Petri dishes were sterilized under UV light for 15 min prior to use. Subsequently, 7 mL of each aqueous matrix (distilled water, industrial wastewater, and river water) collected at 0, 24, and 48 h of treatment were added to the dishes. Five romaine lettuce seeds, previously washed with a 1% H_2_O_2_ solution, were placed in each dish, and all assays were performed in triplicate. Glyphosate was used as a positive control, while distilled water served as a negative control. The dishes were incubated under natural sunlight conditions, avoiding excessive exposure. After four days, root length was measured, and the relative growth index (RGI) was calculated (Text S9).

### 2.9. Analytical Techniques

Hg(II) concentrations were determined using a direct mercury analyzer (Lumex RA-915 LAB) in accordance with EPA Method 7473 [15]. Samples were filtered through 0.22 µm membranes and analyzed by thermal decomposition at 800 °C, followed by atomic absorption spectrometry with Zeeman background correction at 253.7 nm. Calibration was performed using a certified Hg(NO_3_)_2_ standard (R^2^ = 0.9990), and the limit of quantification was 0.0036 µg mL^−1^.

## 3. Results and Discussion

### 3.1. Characterization of Adsorbent Materials Prepared from Rice Husk

**Figure 1:**
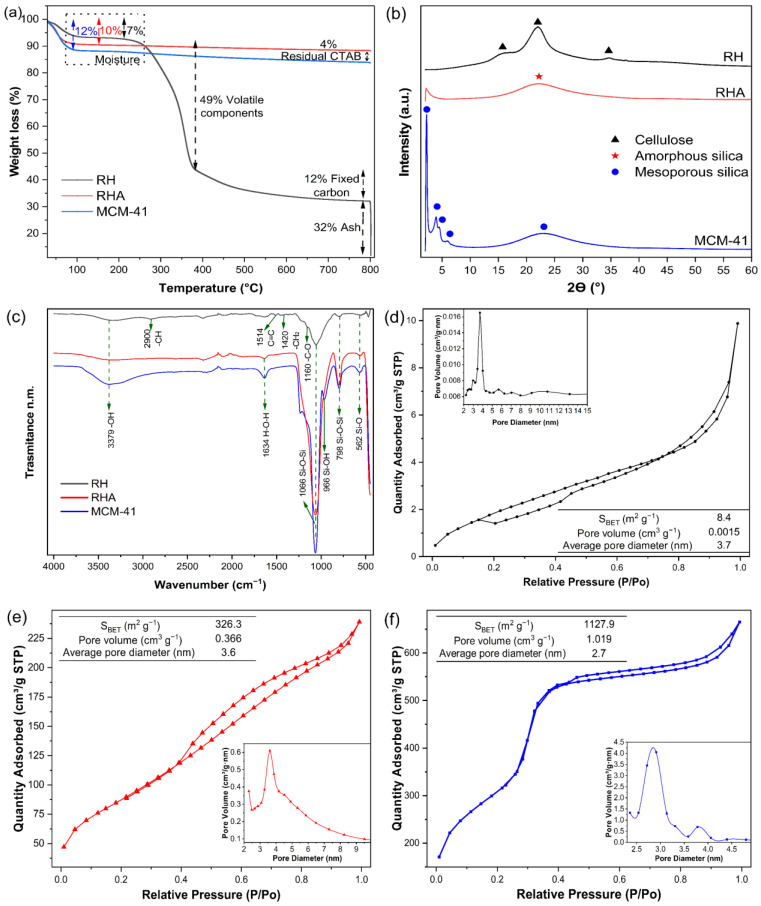
Physicochemical characterization of the adsorbent materials: (**a**) TGA, (**b**) XRD, (**c**) FTIR, N_2_ adsorption–desorption isotherms with porosity characteristics and inset pore size distribution for (**d**) RH, (**e**) RHA, and (**f**) MCM-41.

The physicochemical properties of RH, RHA, and MCM-41 were investigated to elucidate their structural, thermal, and surface characteristics, as well as their potential influence on Hg(II) adsorption. Thermogravimetric analysis (TGA) (Figure 1a) revealed distinct thermal behaviors among the materials. RH exhibited multiple weight loss stages, including an initial loss (7%) around 100 °C, due to physically adsorbed water, followed by a major decomposition event between 220 and 380 °C (~49%) associated with the thermal degradation of hemicellulose and cellulose. A further mass loss (~12%) was associated with the removal of carbonaceous materials. At 800 °C, a residual mass of ~32 wt.% remained, mainly composed of silica (SiO_2_) [16]. In contrast, RHA and MCM-41 exhibit negligible weight losses above 100 °C, indicating their high thermal stability, which is attributed to the predominantly inorganic compositions, mainly amorphous silica in the case of RHA [17] and silanol groups in MCM-41 [18]. A minor weight loss (~4%) observed for MCM-41 above 250 °C suggests the presence of residual template (CTAB). These results confirm the successful transformation of the biomass into thermally stable silica-based materials [19].

XRD patterns (Figure 1b) further confirmed the structural evolution of the materials. RH shows diffraction peaks at 16.09°, 22.06°, and 34.84°, which are associated with cellulose, in agreement with reports indicating that cellulose contents reaching 35% in rice husk [20]. RHA exhibits a broad diffraction band centered around 22°, indicating the presence of amorphous silica, consistent with its reported SiO_2_ content of approximately 95% [21]. In contrast, MCM-41 showed well-defined low-angle reflections at 2.21°, 3.98°, 4.61°, and 6.02° corresponding to the (100), (110), (200), and (210) planes, respectively, confirming the formation of a hexagonal mesoporous structure [19,22]. The calculated interplanar spacing (d_hkl_) values (Table S3) were 0.02 Å for RH and ~6 Å for RHA. In the case of MCM-41, this material exhibited a spacing of ~40 Å for the (100) plane, which is associated with the templated synthesis and the formation of an ordered mesoporous framework. This structural organization is expected to facilitate Hg(II) diffusion and enhance adsorption.

FTIR spectra (Figure 1c) shows that RH exhibited bands related to organic components, including C–H stretching (~2900 cm^−1^), CH_2_ bending (~1420 cm^−1^), aromatic C=C vibrations (~1514 cm^−1^), and C–O stretching (~1160 cm^−1^), associated with lignocellulosic and hemicellulose structure [23,24]. However, RH, RHA, and MCM-41 showed similar bands around 3379 cm^−1^, attributed to –OH stretching vibrations from alcohol groups and adsorbed water, which is further supported by the band observed at 1634 cm^−1^ corresponding to the H–O–H bending vibration. In contrast, RHA and MCM-41 showed bands associated with silica, including Si–O–Si asymmetric (~1066 cm^−1^) and symmetric (798 cm^−1^) stretching, as well as Si–O–Si bending (~562 cm^−1^). A characteristic band at 966 cm^−1^, attributed to Si–OH groups, was observed only in MCM-41, indicating the presence of surface silanol groups. The higher intensity of silica-related bands in RHA and MCM-41 confirms the enrichment of silica during thermal and chemical treatments. The presence of silanol groups in MCM-41 is particularly relevant, as they can act as active sites for Hg(II) adsorption through coordination interactions.

The point of zero charge (pH_PZC_) values (Figure S1) was determined as 5.12, 3.75, and 2.96 for RH, RHA, and MCM-41, respectively, indicating a progressive increase in surface acidity. The lower pH_PZC_ of MCM-41 is associated with the presence of Si–OH groups [25], as corroborated by FTIR analysis (Figure 1c).

Nitrogen adsorption–desorption isotherms (Figure 1d–f) revealed significant differences in the textural properties of the materials. RH and RHA exhibited type II isotherms, characteristic of non-porous or weakly porous materials [26], whereas MCM-41 exhibits a type IV isotherm, indicative of a mesoporous structure [27]. Furthermore, the isotherms for RHA and MCM-41 display an H4-type hysteresis loop, commonly associated with narrow slit-like pores [28], in contrast, RH showed an H3-type hysteresis loop, characteristic of wedge-shaped pores [29]. The low porosity of RH and RHA is confirmed by their small total pore volumes (≤0.366 cm^3^ g^−1^) compared with that of MCM-41 (1.019 cm^3^ g^−1^). Although RH and RHA are weakly porous materials, they still exhibit a pore size distribution with an average pore diameter in the 2–50 nm range, indicating the presence of mesopores according to the IUPAC classification [30], like MCM-41 (Inset Figure 1d–f). In addition to its well-defined mesoporous structure, MCM-41 stands out due to its high specific surface area (1227.9 m^2^ g^−1^), which is significantly higher than that of RH (8.4 m^2^ g^−1^) and RHA (326.3 m^2^ g^−1^). Notably, these surface area values are higher than or comparable to those reported for similar materials in the literature, such as RH (7.1 m^2^ g^−1^ [31], 5.5 m^2^ g^−1^ [32]), RHA (20–270 m^2^ g^−1^ [33], 22 m^2^ g^−1^ [34]), and MCM-41 (1034 m^2^ g^−1^ [35], 971 m^2^ g^−1^ [36]).

**Figure 2:**
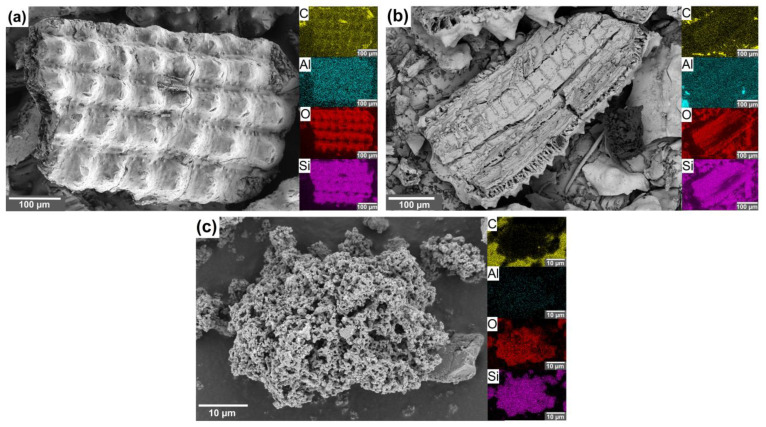
SEM micrographs for (**a**) RH, (**b**) RHA, (**c**) MCM-41.

SEM images (Figure 2a–c) revealed notable morphological differences among the materials. RH exhibited an ordered surface topography, with parallel structure, whereas RHA showed a more heterogeneous morphology with elongated fragments and structural features derived from thermal decomposition [37]. MCM-41 consisted of irregular aggregates with granular domains and evident porosity, consistent with its mesoporous nature. EDS analysis (Table S4) and elemental mapping (Figure 2a–c) confirmed the presence of O and Si in all materials, while C was mainly detected in RH and MCM-41. Minor amounts of Al were also detected. These findings were corroborated by XRF analysis (Table S5), confirming the high silica content of the materials.

The applied thermal and chemical treatments altered the structural, chemical, and textural properties of rice husk-derived materials. Specifically, MCM-41 exhibited a highly ordered mesoporous structure, elevated surface area, and a high concentration of silanol groups, characteristics expected to enhance Hg(II) adsorption. These findings establish a good foundation for interpreting the adsorption performance of these materials in aqueous systems.

### 3.2. Efficiency of Hg(II) Adsorption and Kinetics of the Process Using Rice Husk-Based Adsorbents

**Figure 3:**
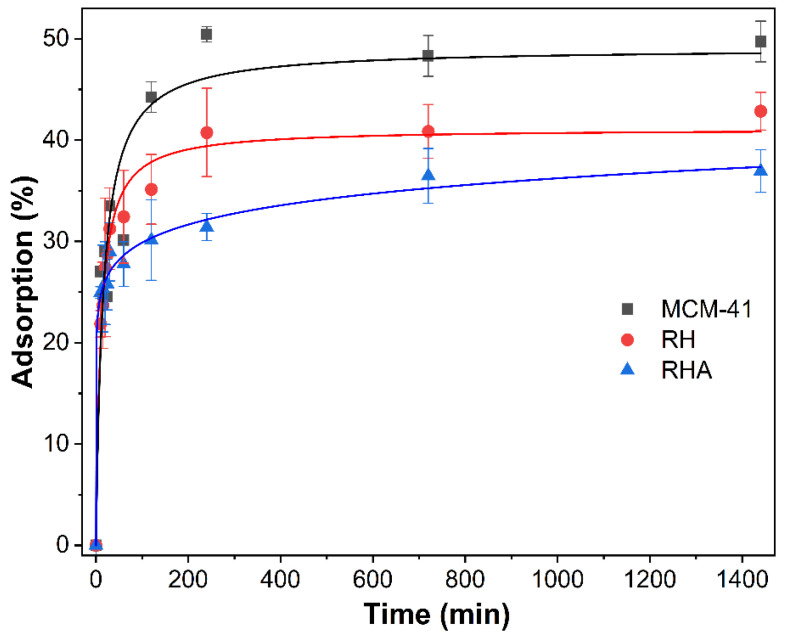
Removal of Hg(II) present in distilled water using MCM-41, RH, and RHA as adsorbents. Conditions: pH 6.8, initial concentration of Hg(II) 0.90 mg L^−1^, stirring rate 270 rpm, adsorbent dose 0.5 g L^−1^, temperature 25 °C.

Figure 3 presents the adsorption performance of RH, RHA, and MCM-41 for Hg(II) removal. The removal efficiency follows the order MCM-41 > RH > RHA, demonstrating the superior performance of the mesoporous material. This trend is attributed to the surface charge properties. Based on the pH_PZC_ values (Figure S1), all materials exhibit a negatively charged surface under the experimental conditions (pH 6.8). MCM-41, with the lowest pH_PZC_, exhibits a higher degree of surface deprotonation, and a greater density of negatively charged sites. This characteristic is linked to the presence of silanol groups (Si–OH), as confirmed by FTIR analysis (Figure 1c), thereby enhancing electrostatic interactions and coordination with Hg(II). Beyond surface charge effects, the superior performance of MCM-41 is also influenced by its textural properties. As discussed in Section 3.1, MCM-41 exhibits a highly ordered mesoporous structure, a large specific surface area, and a high pore volume. These features collectively increase the availability and accessibility of active sites, thereby facilitating Hg(II) diffusion and adsorption.

The adsorption profiles (Figure 3) show that equilibrium was reached at approximately 720 min for all materials. Extending the contact time to 1440 min did not result in significant desorption, indicating strong interactions between Hg(II) and the adsorbent surfaces and suggesting stable adsorption processes.

To further elucidate the adsorption mechanism, kinetic data were fitted to pseudo-first-order and pseudo-second-order models (Figure S2) with the corresponding parameters summarized in Table 1. For all materials, the pseudo-second-order model yielded a better fit, as indicated by higher correlation coefficients (R^2^), lower average percentage error (APE), and closer agreement between calculated and experimental adsorption capacities (q_e_,cal and q_e_,exp). These results suggest that the adsorption process is governed by interactions involving the availability of active sites [38]. MCM-41 exhibited higher adsorption capacities and kinetic constants (k_2_) compared to RH and RHA, indicating faster and more effective Hg(II) uptake. These findings confirm that both surface chemistry and textural properties are critical determinants of adsorption performance. Accordingly, RH and RHA exhibited poorer adsorption dynamics, likely due to their physicochemical properties, as discussed in this section. Therefore, they were not considered for further detailed analysis. Given its superior efficiency and favorable kinetic behavior, MCM-41 was selected as the most suitable adsorbent for further evaluation. Thus, the next section evaluates the influence of operational parameters on its adsorption performance to identify optimal conditions and to better understand the adsorption process under environmentally relevant scenarios.

### 3.3. Effects of Operational Parameters on Hg(II) Removal

**Figure 4:**
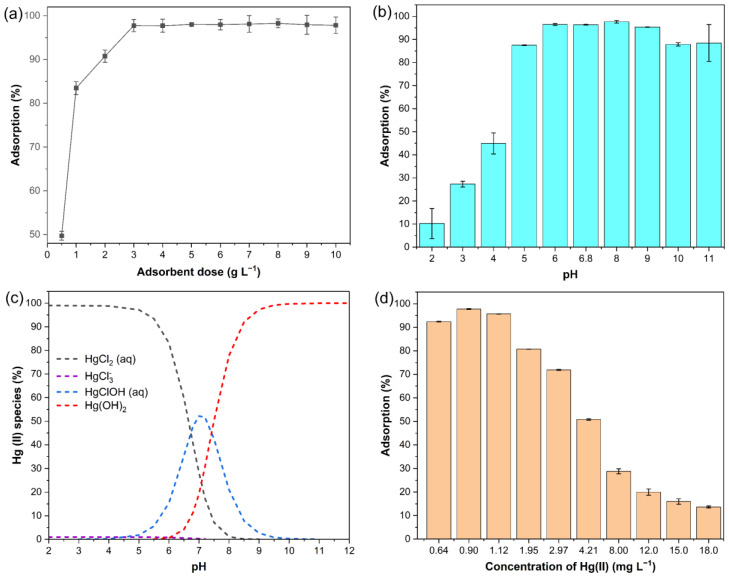
Influence of key variables on the adsorption of Hg(II) onto MCM-41: (**a**) Effect of adsorbent dosage on percent Hg(II) removal. Conditions: pH 6.8, initial concentration of Hg(II) 0.90 mg L^−1^, stirring rate 270 rpm, time 24 h, temperature 25 °C. (**b**) Effect of pH on the adsorption efficiency of Hg(II). Conditions: initial concentration of Hg(II) 0.90 mg L^−1^, stirring rate 270 rpm, time 24 h, adsorbent dose 3.0 g L^−1,^ temperature 25 °C. (**c**) Distribution of Hg(II) species diagram using the Visual MINTEQ program. Conditions: [Hg] = 0.90 mg L^−1^, [HCl] = 0.001 mol L^−1^. (**d**) Effect of initial Hg(II) concentration on the percent adsorption. Conditions: pH 6.8, stirring rate 270 rpm, time 24 h, adsorbent dose 3.0 g L^−1,^ temperature 25 °C.

The influence of key operational parameters on Hg(II) removal by MCM-41 was evaluated to optimize the adsorption process and elucidate the underlying mechanisms. Figure 4a presents the effect of adsorbent dosage. Hg(II) removal increased markedly with higher MCM-41 dosage, from approximately 50% at 0.5 g L^−1^ to a maximum of 98% at 3.0 g L^−1^. This enhancement is attributed to the greater availability of active adsorption sites and increased surface area, which enhance adsorbent–adsorbate interactions [39]. Beyond this dosage, removal efficiency remained nearly constant, indicating that the availability of Hg(II) in solution became the limiting factor. Thus, 3.0 g L^−1^ was determined to be the optimal dosage, balancing removal efficiency and resource utilization.

Figure 4b shows the effect of solution pH on Hg(II) removal efficiency, along with the corresponding speciation diagram (Visual MINTEQ simulations) (Figure 4c). Under highly acidic conditions (pH 2), removal efficiencies were low due to the positively charged surface of MCM-41, which inhibits Hg(II) adsorption. In the pH range of 3 to 4, adsorption remained limited due to the predominance of HgCl_2_ species and competition with protons (H^+^) for available adsorption sites [40]. As pH increased to 5, reduced proton competition resulted in a moderate improvement in adsorption. At near-neutral pH values (6–8), Hg(II) predominantly exists as HgClOH, which interacts effectively with deprotonated oxygen-containing surface groups (Si–O^−^), promoting surface complexation and enhancing adsorption efficiency [41,42]. In contrast, at pH ≥ 9, the formation of Hg(OH)_2_ leads to precipitation, which limits adsorption and reduces the practical applicability of the process [43]. These results indicate that efficient Hg(II) removal can be achieved at natural pH (≈6.8), eliminating the need for pH adjustment and enhancing process feasibility.

Figure 4d shows the effect of the initial Hg(II) concentration. The highest removal efficiency (98%) was achieved at an initial Hg(II) concentration of 0.90 mg L^−1^. As the initial concentration increased, the removal percentage decreased to 14% at 18.0 mg L^−1^ of Hg(II). This behavior is attributed to the progressive saturation of available adsorption sites at higher pollutant concentrations. At low concentrations, sufficient active sites are available to achieve high removal efficiency, whereas at higher concentrations, excess Hg(II) remains in solution due to site limitations [44]. Despite this decrease, MCM-41 maintained substantial removal efficiency across a broad concentration range, supporting its potential applicability under variable contamination conditions. These results support further evaluation of its adsorption capacity through equilibrium and thermodynamic analyses.

### 3.4. Isotherm Modeling and Thermodynamic Assessment of Adsorption

**Figure 5:**
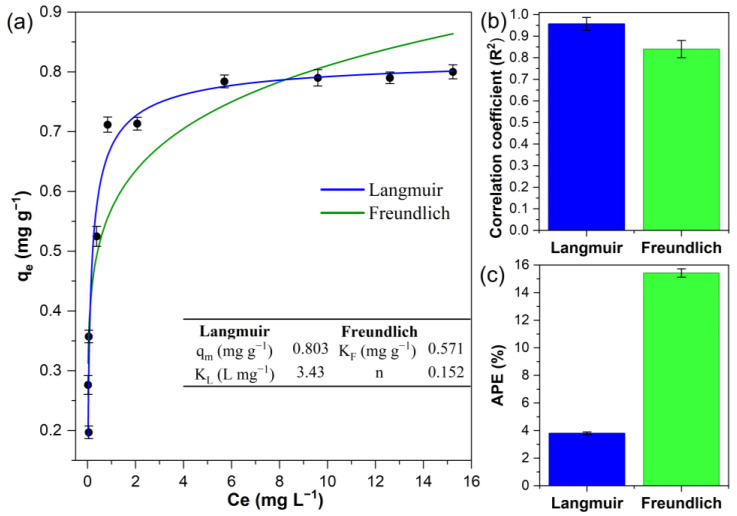
Analysis of adsorption isotherms of Hg(II) using MCM-41. (**a**) Adjustment to the isotherms of Langmuir and Freundlich. Conditions: pH 6.8, stirring rate 270 rpm, time 24 h, adsorbent dose 3.0 g L^−1^, initial concentration Hg(II) in the range of 0.64–18.0 mg L^−1^, temperature 25 °C. (**b**) Correlation coefficient (R^2^) and (**c**) APE (%) results.

Adsorption isotherms were employed to characterize the equilibrium behavior of Hg(II) on MCM-41 and to estimate its adsorption capacity. The experimental data were analyzed using the Langmuir and Freundlich models (Figure 5a). The Langmuir model provided the best fit, yielding a maximum Hg(II) adsorption capacity (qₘ) of 0.80 mg g^−1^. The high Langmuir constant (K_L_) indicates a strong affinity between Hg(II) and the adsorbent surface. Additionally, the Freundlich model showed a constant (K_F_) of 0.57 mg g^−1^ and an adsorption intensity parameter (*n*) lower than 1, suggesting less favorable adsorption. However, a better fit was observed for the Langmuir isotherm, supported by the higher correlation coefficient (R^2^) (Figure 5b) and lower average percentage error (%APE) (Figure 5c). Collectively, these results suggest that Hg(II) adsorption on MCM-41 predominantly occurs as monolayer coverage over a relatively homogeneous surface.

The adsorption capacity (q_m_) of MCM-41 determined in this study is comparable to those reported for similar materials, such as mordenite (0.76 mg g^−1^) [45] and clay-based zeolites (0.83 mg g^−1^) [46]. However, it is lower than the values reported for modified or optimized MCM-41 systems, including unmodified (56.48 mg g^−1^) [47] and functionalized forms such as NH_2_-MCM-41 (63.3 mg g^−1^) [48] and ZnCl_2_-MCM-41 (204 mg g^−1^) [49]. The discrepancy between the performance of MCM-41 in this study and the higher values reported in the literature can be primarily attributable to differences in experimental conditions. In several previous studies, initial Hg(II) concentrations reach up to 1000 mg L^−1^, adsorbent dosages are relatively high (>3.5 g L^−1^), and pH values (2–5) are not representative of Hg speciation in natural waters [50,51]. Such conditions may lead to an overestimation of adsorption performance rather than reflecting practical applicability. Moreover, the higher adsorption capacities reported in the research are often associated with surface functionalization, which increases the density of active sites and strengthens metal–adsorbent interactions. In contrast, MCM-41 stands out as it was synthesized via a simple route from rice husk without chemical functionalization, minimizing reagent use and supporting a greener synthesis approach.

The effect of temperature on adsorption performance (Figure S3) showed a decrease in Hg(II) uptake with increasing temperature, indicating an exothermic adsorption process [52]. Thermodynamic parameters (ΔG°, ΔH°, and ΔS°) were calculated (Table 2 and Figure S4) to further elucidate the adsorption mechanism. The negative value of ΔH° confirms the exothermic nature of the process, and its magnitude (<80 kJ mol^−1^) suggests that Hg(II) adsorption onto MCM-41 occurs predominantly through physisorption [53]. Negative ΔG° values indicate that the process is spontaneous under the conditions studied. The positive ΔS° values indicate increased randomness at the solid–liquid interface, which may result from the release of water molecules and structural rearrangements during adsorption. Collectively, these findings demonstrate that Hg(II) ions adsorption onto MCM-41 is a spontaneous and exothermic process, primarily driven by physical interactions, with additional contributions from surface complexation mechanisms as suggested by FTIR analysis.

### 3.5. Mechanistic Proposal

**Figure 6:**
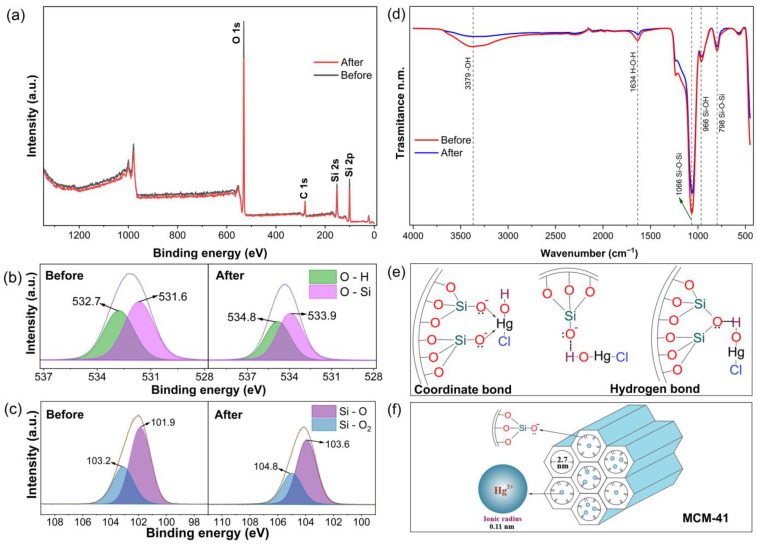
Proposed mechanisms of Hg(II) adsorption onto MCM-41 using a concentration of 0.90 mg L^−1^ of Hg(II), adsorbent dose 3.0 g L^−1^, stirring rate 270 rpm, and pH 6.8. (**a**) XPS of MCM-41. (**b**) Deconvoluted O 1s and (**c**) Si 2p before and after Hg(II) adsorption. (**d**) FTIR spectra of MCM-41 before and after Hg(II) adsorption. (**e**) Interaction of Hg(II) with –Si–O– and –Si–O–Si groups. (**f**) Contribution of the internal structure of the material to the adsorption process.

A mechanistic interpretation of Hg(II) removal by MCM-41 was proposed based on adsorption performance and complementary analyses, utilizing XPS, XRF, and FTIR. The XPS survey spectrum shows the characteristic signals of O 1s, C 1s, and Si 2p (Figure 6a). After Hg(II) adsorption, a decrease in the intensity of these signals is observed for O and Si, which can be attributed to slight changes in the surface electronic density of MCM-41 upon interaction with the metal species. In contrast, no significant variations are detected in the C 1s signal associated with residual traces of the CTAB surfactant used during the synthesis process.

Additionally, shifts in the binding energy of the O 1s and Si 2p levels are observed (Figure 6b,c). These shifts indicate modifications in the chemical environment of silanol (Si–OH) and siloxane (Si–O–Si) groups, suggesting that Hg(II) adsorption may occur through electrostatic interactions and/or charge transfer with oxygen atoms in the silica framework. However, no Hg(II) signal is directly detected in the XPS spectra. This absence can be explained by the surface-sensitive nature of XPS, which probes only a few nanometers in depth, and by the low Hg(II) concentration after adsorption, which likely results in a surface content below the typical detection limit (~0.1 at%). Therefore, Hg(II) species are likely located predominantly within the pore structure of MCM-41 rather than on the external surface. To overcome this limitation, the analysis was complemented with XRF (Table S6), a bulk-sensitive technique that confirms the presence of Hg(II) at traces levels in the material after adsorption, with a content of approximately 0.01 wt%.

In addition, FTIR spectra obtained before and after adsorption (Figure 6d) supported the XPS results. Following Hg(II) adsorption, the intensity of bands associated with –OH (3370 cm^−1^), H–O–H (1634 cm^−1^), and Si–OH (966 cm^−1^) groups decreased, indicating the participation of surface silanol groups in Hg(II) binding. Under the experimental pH conditions, silanol groups (pKa ≈ 4.5) [54] are deprotonated (–Si–O^−^), which enables them to function as Lewis bases. These sites interact with Hg(II) species, particularly HgClOH, via coordination and surface complexation mechanisms, with Hg(II) acting as a soft Lewis acid [55]. Additionally, –Si–O^−^ groups may form bonds with the hydrogen present in HgClOH, as illustrated in Figure 6e. Slight decreases in the bands at 1066 and 798 cm^−1^, associated with –Si–O–Si vibrations, suggest that the silica framework may also participate indirectly in the adsorption process, potentially through hydrogen bonding with HgClOH.

The textural properties of MCM-41 also play a significant role in the adsorption mechanism. The material possesses a well-defined mesoporous structure with an average pore diameter of 2.7 nm (inset Figure 1f). This structure facilitates the diffusion of Hg(II) species (ionic radius of 0.11 nm) into the internal structure of material (Figure 6f), thereby enhancing the accessibility of active sites and contributing to overall adsorption efficiency. In summary, Hg(II) removal by MCM-41 is governed by both surface complexation involving silanol groups and efficient diffusion within the mesoporous structure. These observations are consistent with those discussed in Section 3.2.

### 3.6. Adsorbent Reusability and Application in Complex Matrices

**Figure 7:**
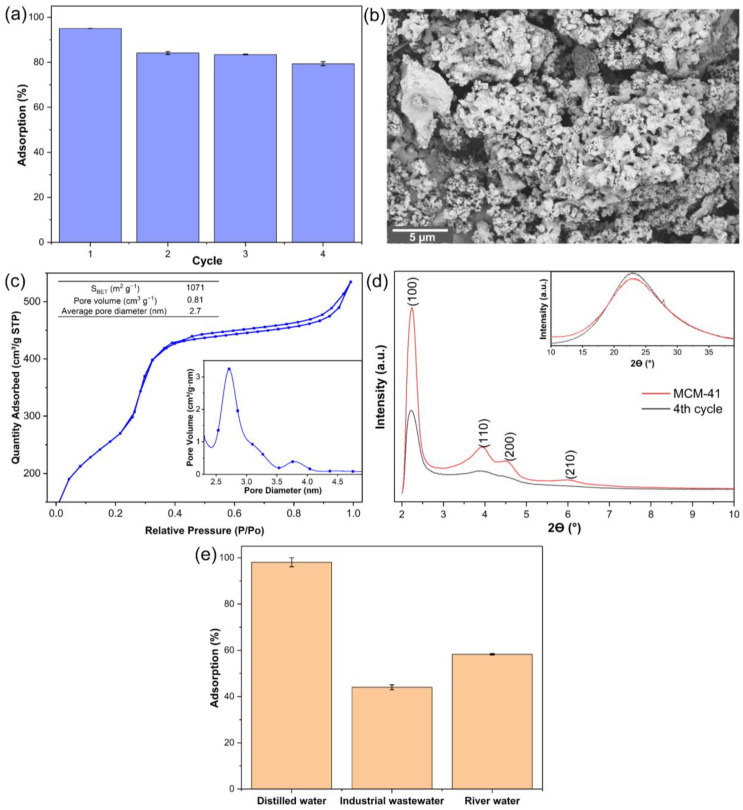
Reusability of MCM-41 for Hg(II) removal, post-reuse characterization, and application complex matrices. (**a**) Reuse of MCM-41 during four cycles. Analysis of the material after adsorption by (**b**) micrograph SEM, (**c**) nitrogen adsorption–desorption isotherm with porosity characteristics, (**d**) XRD analysis, and (**e**) MCM-41 in the removal of Hg(II) in industrial wastewater and river water. Conditions: initial concentration of Hg(II) 0.90 mg L^−1^, stirring rate 270 rpm, adsorbent dose 3.0 g L^−1^, pH 6.80, and temperature 25 °C.

The reusability of MCM-41 was assessed over four consecutive adsorption–desorption cycles to evaluate its stability for practical applications (Figure 7a). The material maintained a removal efficiency of approximately 80% after the fourth cycle, indicating that a substantial proportion of active sites remained accessible and the adsorbent retained its functionality [56]. SEM analysis (Figure 7b) confirmed that the aggregated and porous morphology of MCM-41 was largely retained after reuse. The average pore diameter also remained nearly unchanged (Figure 7c, inset), indicating preservation of the mesoporous framework. However, reductions in specific surface area (from 1227.9 to 1071 m^2^ g^−1^) and pore volume (to 0.81 cm^3^ g^−1^) (Figure 7c) were observed, suggesting partial pore blockage likely caused by the accumulation of residual species within the pore network. This phenomenon may limit the accessibility of active sites and contribute to the gradual decline in adsorption capacity [57]. XRD analysis (Figure 7d) revealed a decrease in the intensity of the (100) reflection and reduced definition of the (110) and (200) peaks after repeated cycles, while the (210) reflection was no longer observed (Table S7). These changes suggest a partial loss of structural ordering within the mesoporous framework. However, the persistence of the broad peak at 22° (inset Figure 7d), confirms that the material maintains its silica-based composition. FTIR analysis also showed changes in the intensity of Si–O–Si and Si–OH bands (Figure S5), supporting the involvement of these functional groups in the adsorption process and indicating their gradual modification during reuse. Despite these changes, the results demonstrate that MCM-41 maintains satisfactory performance and structural integrity after multiple cycles, supporting its potential for repeated use.

The applicability of MCM-41 was further evaluated in complex aqueous matrices, such as synthetic industrial wastewater and real river water, as presented in Figure 7e. Compared to distilled water, Hg(II) removal decreased 45% in industrial water and 58% in river water. This reduction is attributed to the presence of competing ions that occupy active sites and hinder Hg(II) adsorption. Elemental analysis (Table S8) indicated the presence of other metal ions, such as Cd^2+^ and Al^3+^, whose concentrations decreased after treatment. These results suggest that MCM-41 can interact with multiple metal species. This competitive adsorption behavior accounts for the reduced Hg(II) removal efficiency in complex matrices. Nevertheless, MCM-41 still demonstrates strong potential for practical applications. These findings suggest that its selectivity and overall performance in multi-component systems could be enhanced through targeted optimization strategies, such as surface modification or process parameter adjustment. Moreover, the observed competitive interactions indicate its capability to simultaneously remove multiple metal ions, thereby expanding its applicability for the treatment of complex contaminated waters.

### 3.7. Ecotoxicological, Microbiological and Phytotoxic Assessment in Treated Aqueous Matrices

#### 3.7.1. Ecotoxicity Analysis

**Figure 8:**
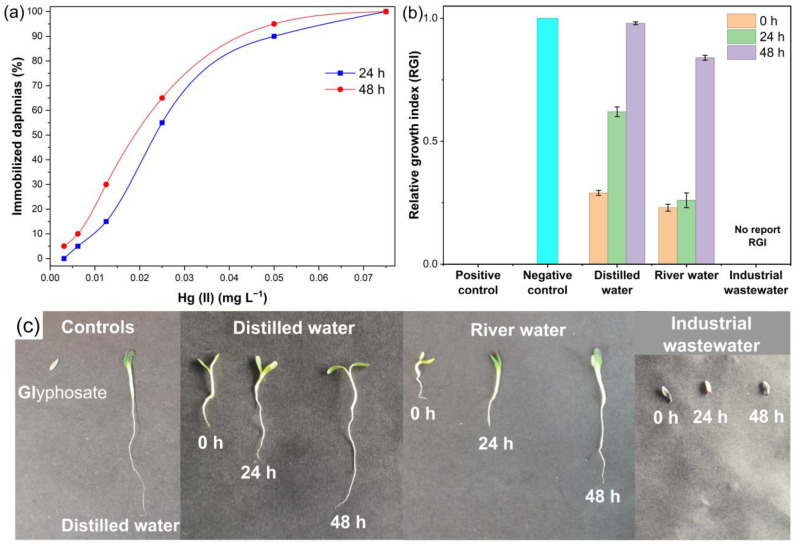
Ecotoxicological and phytotoxicity assessment. (**a**) Immobility of *Daphnia magna*. Conditions: pH 6.5, temperature 22 °C, distilled water supplemented with saline solution (Table S1) using Hg(II) concentrations ranging from 0.0031 to 0.075 mg L^−1^. (**b**) Relative growth index. (**c**) Growth response in *Lactuca sativa.* Conditions: temperature 25 °C, initial concentration of Hg(II) 0.90 mg L^−1^, adsorbent dose 3.0 g L^−1^, pH 6.8.

After Hg(II) removal using MCM-41, the treated water was assessed to determine whether contaminant reduction corresponded to a decrease in biological toxicity. Acute ecotoxicity tests utilized *Daphnia magna*, a sensitive and widely recognized bioindicator of aquatic contamination [58]. The results indicated complete immobilization (100%) of organisms after 24 h of exposure in all treated aqueous matrices, including distilled water, industrial wastewater, and river water (Figure S6). To better understand this behavior, the median effective concentration (EC_50_) of Hg(II) was determined in distilled water. As shown in Figure 8a, immobilization increased with both exposure time and Hg(II) concentration, reaching complete immobilization at 24 h and 48 h. Probit analysis (Figure S7) yielded EC_50_ values of 0.0220 ± 0.0026 mg L^−1^ at 24 h and 0.0158 ± 0.0019 mg L^−1^ at 48 h, indicating increased toxicity with prolonged exposure time.

These findings clarify the observed immobilization in treated samples. In distilled water, despite the high removal efficiency of MCM-41, a residual Hg(II) concentration of approximately 0.018 mg L^−1^ remained (Figure 7e), which is close to the determined EC_50_ values. This demonstrates that even low residual concentrations can induce significant toxic effects. In more complex matrices, such as industrial wastewater and river water, the residual concentrations were significantly higher, reaching 0.495 mg L^−1^ (55% non-removal) and 0.378 mg L^−1^ (42% non-removal), respectively. These concentrations exceed the EC_50_ thresholds, accounting for the complete immobilization observed under these conditions. These findings demonstrate that although MCM-41 effectively reduces Hg(II) concentrations, residual levels can still pose a significant ecotoxicological risk. This underscores the need to combine removal efficiency with toxicity assessment when evaluating the environmental performance of water treatment technologies.

#### 3.7.2. Microbiological Assessment

Microbiological analyses were performed to assess the quality of treated water across various matrices, and the results are presented in Table S9. The initial assessment of *E. coli* and total coliforms in the original (undoped) samples revealed that only river water exhibited microbial contamination, with 30 CFU/100 mL of *E. coli* and 720 CFU/100 mL for total coliforms. Following the addition of Hg(II), no bacterial growth was detected in either untreated or MCM-41-treated samples, which inhibits bacterial growth even at relatively low concentrations. This observation is consistent with previous reports showing that Hg(II) toxicity effectively inhibits the growth of *E. coli* and total coliforms [59].

To further examine the potential contribution of the adsorbent, an additional experiment was performed in which MCM-41 was placed in contact with river water in the absence of Hg(II). Under these conditions, neither *E. coli* nor total coliforms were detected, indicating that MCM-41 may possess antimicrobial activity. This effect may be related to the surface properties of silica-based materials, particularly the presence of silanol groups (Si–O^−^), which can enhance interactions with bacterial membranes and disrupt cellular integrity [60].

However, the absence of microorganisms in Hg(II)-containing samples is primarily attributed to the toxic effects of the contaminant rather than to genuine improvement in water quality. These findings demonstrate that microbiological indicators alone are insufficient for assessing water safety when toxic pollutants are present. This behavior suggests that while MCM-41 may contribute to microbial inhibition, the observed effects are predominantly due to Hg(II) toxicity.

Overall, these findings demonstrate that ecotoxicological and microbiological assessments contribute to ensuring environmental safety, as they allow identifying that, although MCM-41 reduces Hg(II) concentrations, the treated water still exhibits significant biological effects. In this sense, these evaluations make it possible to determine that the treated water under these conditions is not suitable for environmental discharge or reuse without additional treatment, thereby supporting the safe management of contaminated water. These results underscore the need for complementary assessments, such as phytotoxicity testing, to determine the suitability of treated water for safe environmental applications.

#### 3.7.3. Evaluation of Phytotoxic Effects in Lactuca Sativa

Phytotoxicity of treated aqueous matrices was assessed using *Lactuca sativa* seed germination assays, with the relative root growth index (RGI) determined. Distilled water, industrial wastewater, and river water samples treated with MCM-41 were analyzed (Figure 8b). Glyphosate, used as a positive control, completely inhibited germination, confirming the assay’s sensitivity. Distilled water, serving as a negative control, resulted in normal germination, validating seed viability. In contrast, distilled and river water spiked with Hg(II) at 0 h exhibited lower RGI values, confirming the contaminant’s phytotoxic effect. Mercury exposure inhibits key enzymes, including amylase and protease, which are essential for seed germination [61]. Following treatment with MCM-41, RGI increased progressively over time, reaching a maximum at 48 h. According to the RGI classification (Table S10), values of 0.98 for distilled water and 0.84 for river water indicate no significant phytotoxic effects. These results suggest that Hg(II) toxicity was effectively mitigated under these conditions. In contrast, industrial wastewater remained highly toxic after treatment, as no germination was observed at any of the evaluated time points. This persistent toxicity may be attributed to the presence of multiple elements, such as Al, Ni, S, and Fe (Table S8), which can inhibit plant development. Moreover, synergistic effects among these elements may further suppress germination and growth [62].

Figure 8c illustrates the germination process in the different matrices. No germination occurred in the glyphosate treatment, whereas plants grown in distilled water reached an average root length of 3.67 cm. In distilled water and river water contaminated with Hg(II), germination occurred, but plants exhibited reduced root length and chlorosis, indicating physiological stress caused by the presence of heavy metal [63]. Reduced stem growth reflects disruption of physiological processes, while leaf yellowing results from interference with chlorophyll biosynthesis [64]. Treatment with MCM-41 progressively mitigated these adverse effects. Partial recovery was observed after 24 h in distilled water and 48 h in river water, confirming the effectiveness of the adsorbent in reducing Hg(II)-induced phytotoxicity. However, the absence of germination in industrial wastewater underscores the limitations of this process in complex matrices. These findings demonstrate that MCM-41 effectively reduces Hg(II) phytotoxicity in river water, supporting its potential application to improve water quality for irrigation. However, additional treatment strategies are required for highly complex matrices such as industrial wastewater.

## 4. Conclusions

The characterization analysis indicated that RH is rich in lignocellulosic components, whereas RHA is primarily composed of amorphous silica. In contrast, MCM-41 exhibited superior textural and surface properties, including a well-defined mesoporous structure, high surface area, large interplanar spacing, and abundant silanol groups, consistent with materials from the M41S family. These characteristics significantly enhanced its adsorption performance. All materials demonstrated the capacity to remove Hg(II) following a pseudo-second-order kinetic model, suggesting that adsorption is governed by the availability of active sites. Among these, MCM-41 exhibited the highest adsorption rate and removal efficiency, achieving up to 98% under optimal conditions (pH 6.8, 3.0 g L^−1^, and an initial Hg(II) concentration of 0.90 mg L^−1^). The adsorption process was spontaneous and exothermic, primarily driven by surface complexation involving deprotonated silanol groups, with additional contributions from hydrogen bonding and diffusion within the material’s internal structure.

MCM-41 also exhibited good reusability, maintaining up to 80% Hg(II) removal efficiency after four cycles, although some performance loss occurred due to partial structural degradation and pore blockage. In complex water matrices such as river water and industrial wastewater, removal efficiency decreased due to the competitive effects of coexisting ions. Nevertheless, the material retained a relevant adsorption capacity under realistic conditions. Ecotoxicological and microbiological assessments indicated that residual Hg(II) concentrations were sufficient to induce biological effects, including complete immobilization of *Daphnia magna* and inhibition of bacterial growth. These results demonstrate that contaminant removal alone does not guarantee environmental safety. In contrast, phytotoxicity assays showed partial recovery of *Lactuca sativa* root growth in treated river water, suggesting potential for crop irrigation applications. However, persistent toxicity in industrial wastewater underscores the need for additional treatment strategies to mitigate it and promote safe and sustainable applications.

## Figures and Tables

**Table 1:** Kinetic parameters of the removal of Hg(II) from distilled water. Conditions: pH 6.8, initial concentration of Hg(II) 0.90 mg L^−1^, stirring rate 270 rpm, adsorbent dose 0.5 g L^−1^, temperature 25 °C.

Adsorbent	Pseudo-First Order Model					Pseudo-Second Order Model			
	q_e,exp_ (mg g^−1^)	q_e,cal_ (mg g^−1^)	k_1_ (min^−1^)	R^2^	APE (%)	q_e,cal_ (mg g^−1^)	k_2_ (mg g^−1^ min^−1^)	R^2^	APE (%)
MCM-41	0.875	0.807	0.0442	0.838	0.807	0.864	0.0753	0.912	0.264
RH	0.760	0.693	0.0624	0.939	0.188	0.730	0.132	0.983	0.124
RHA	0.655	0.562	0.108	0.890	1.12	0.597	0.321	0.947	0.313

**Table 2:** Thermodynamic parameters in the removal of Hg(II) using MCM-41. Conditions: Hg(II) concentration 0.90 mg L^−1^, adsorbent dose 3.0 g L^−1^, stirring rate 270 rpm and pH 6.8.

Thermodynamic Parameters			
Temperature (°C)	∆H (kJ mol^−1^)	∆G (kJ mol^−1^)	∆S (J mol^−1^ K^−1^)
25		−45.74 ± 0.60	
45	−24.11 ± 0.49	−47.19 ± 0.31	72.54 ± 1.56
65		−48.64 ± 0.59	

## Data Availability

The original contributions presented in this study are included in the article/Supplementary Materials. Further inquiries can be directed to the corresponding authors.

## Supplementary Materials

The following supporting information can be downloaded at: https://www.mdpi.com/article/10.3390/nano16110694/s1 Text S1: Interplanar Spacing; Text S2: Adsorption percentage (%); Text S3: Description of the pseudo-first order kinetic model; Text S4: Description of the pseudo-second order kinetic model; Text S5: Average percentage error (APE); Text S6: Description of the Langmuir isotherm; Text S7: Description of the Freundlich isotherm; Text S8: Determination of thermodynamic parameters; Text S9: Relative growth index (RGI); Table S1: Composition of saline solution; Table S2: Composition of the synthetic wastewater; Table S3: Interplanar spacing; Table S4: Elemental composition of adsorbents by energy-dispersive X-ray spectroscopy (EDS); Table S5: Elemental composition of adsorbents by X-ray fluorescence (XRF); Table S6: Elemental composition of MCM-41 after adsorption by X-ray fluorescence (XRF); Table S7: Interplanar spacing post-reuse of MCM-41; Table S8: X-ray fluorescence analysis of industrial wastewater and river water samples; Table S9: Microbiological analysis in Caquetá River. Conditions: Temperature 37 °C, stirring rate 220 rpm, adsorbed dose 3.0 g L^−1^; Table S10: Classification of RGI values; Figure S1: Point of charge zero. Conditions: NaCl 0.1 M, adsorbent dose 1.0 g L^−1^, stirring rate 200 rpm, temperature 25 °C; Figure S2: Kinetics of pseudo-first order and pseudo-second order in the removal of Hg(II) from distilled water using MCM-41, RH and RHA. Conditions: pH 6.8, initial concentration of Hg(II) 0.90 mg L^−1^, stirring rate 270 rpm, adsorbent dose 0.5 g L^−1^, temperature 25 °C; Figure S3: Adsorption capacity at different temperatures. Conditions: pH 6.8, stirring rate 270 rpm, time 12 h, initial concentration of Hg(II) 0.90 mg L^−1^ and adsorbent dose 3.0 g L^−1^; Figure S4: ln Kc vs. 1/T to obtain the thermodynamic parameters in the Hg(II) adsorption. Conditions: Hg(II) concentration 0.90 mg L^−1^, MCM-41 dose 3.0 g L^−1^, pH 6.8, time 12 h, temperature 25–65 °C; Figure S5: FTIR spectra of MCM-41 before and after four adsorption cycles of Hg(II). Conditions: pH 6.8, stirring rate 270 rpm, time 24 h, adsorbent dose 3.0 g L^−1^, initial concentration of Hg(II) 0.90 mg L^−1^; Figure S6: Immobility of *Daphnia magna* in three water matrices. Conditions: pH 6.5, temperature 22 °C, time 24 h; Figure S7: Probits. (a) 24 h, (b) 48 h. Conditions: *Daphnia magna* maintained at 22 °C in distilled water supplemented with saline solution (Table S1) using Hg(II) concentrations ranging from 0.0031 to 0.075 mg L^−1^. References [65,66,67,68,69,70,71,72,73,74,75,76] are cited in the supplementary materials.
