# Supplementary material for: Rice Husk-Derived MCM-41 for Efficient Hg(II) Removal: Performance, Mechanism, and Environmental Safety in Real Water Matrices
Source: Nanomaterials (Basel). 2026 Jun 1;16(11):694. doi: 10.3390/nano16110694 (PMC13257766; doi:10.3390/nano16110694)
Supplement: Supplementary file 1 [file nanomaterials-16-00694-s001.zip › nanomaterials-4308568-supplementary materials.pdf]

nanomaterials MDPI

SUPPLEMENTARY MATERIAL

Rice Husk-Derived MCM-41 for Efficient Hg(II) Removal: Performance, Mechanism, and Environmental Safety in Real Water Matrices

Naren Bocanegra [1], Marcela Paredes-Laverde [2], Nancy Acelas [3,*], Ximena Carolina Pulido [1], Luis Rodríguez [1] and César Jaramillo-Páez [1,*]

[1] Grupo de Investigación en Química Aplicada a Procesos Ecológicos (QUAPE-UT), Facultad de Ciencias, Universidad del Tolima, Ibagué, Colombia; nbocanegram@ut.edu.co (N.B.); xpulido@ut.edu.co (X.C.P.); lfrodriguezh@ut.edu.co (L.R.)
[2] Grupo de Investigación Navarra Medicina, Facultad de Ciencias de la Salud, Fundación Universitaria Navarra-Uninavarra, Neiva, Colombia; marcela.paredes@udea.edu.co
[3] Grupo de Investigación Materiales con Impacto (Mat&mpac), Facultad de Ciencias Básicas, Universidad de Medellín, Medellín, Colombia
* Correspondence: nyacelas@udemedellin.edu.co (N.A.); cajaramillopa@ut.edu.co (C.J.-P.)

Text SM1. Interplanar Spacing

Interplanar spacing is defined as the distance between adjacent planes of atoms in a crystal lattice [1]. The d_{hkl} values were calculated using Bragg's equation [2]:

$$d_{hkl} = \frac{n\lambda}{2\sin\theta} \quad (1)$$

where: d_{hkl}—interplanar distance, n—reflection order, λ—wavelength, θ—angle of reflection

Text SM2. Adsorption percentage (%)

The adsorption percentage (%) was calculated using the following equation:

$$(\%) = \frac{(C_0 - C_e)}{C_0} * 100 \quad (2)$$

Where C_0 is the initial Hg(II) concentration (mg L^{-1}), C_e is the equilibrium Hg(II) concentration (mg L^{-1}).

Text SM3. Description of the pseudo-first order kinetic model

The nonlinear equation describing this model is [3]:

$$q_t = q_e(1 - e^{-k_1 t}) \quad (3)$$

where q_t represents the amount adsorbed at time t (mg·g^{-1}), q_e is the equilibrium adsorption capacity (mg·g^{-1}), and k_1 is the rate constant (min^{-1}).

The determination of the amount adsorbed at equilibrium q_e (mg g^{-1}) was calculated using:

$$q_e = \frac{C_0 - C_e}{m} * V \quad (4)$$

where C_0 is the initial concentration (mg L^{-1}); C_e is the concentration at equilibrium (mg L^{-1}); m is the mass of adsorbent used (g) y V is the volume of the aqueous solution (L).

Text SM4. Description of the pseudo-second order kinetic model

The nonlinear equation describing this model is [4]:

$$q_t = \frac{{q_e}^2 k_2 t}{1 + q_e k_2 t} \quad (5)$$

where k_2 is the pseudo-second-order rate constant (mg·g^{-1}·min^{-1}), t is time (min), q_e is calculated ad described in Text SM3.

Text SM5. Average percentage error (*APE*)

The average percentage error (*APE*) was calculated using the following equation:

$$\text{APE }(\%) = \frac{\sum_{i=1}^{N}\left|\left(\frac{q_{exp} - q_{cal}}{q_{exp}}\right)\right|}{N} * 100 \qquad (6)$$

Where q_{exp} and q_{cal} represent the experimental and calculated equilibrium amounts of Hg(II) adsorbed, respectively, and N denotes the number of measurements. The APE reflects the degree of agreement between experimental data and the model-predicted adsorption values.

Text SM6. Description of the Langmuir isotherm

The Langmuir isotherm, was calculated according to the following equation [5]:

$$q_e = \frac{q_m K_L C_e}{1 + K_L C_e} \quad (7)$$

where C_e (mg L^{-1}) and q_e (mg g^{-1}) represent, respectively, the equilibrium concentration and the amount of adsorbate retained per unit mass of adsorbent; while q_m (mg g^{-1}) corresponds to the maximum adsorption capacity of Hg(II), i.e., the amount necessary to form a complete monolayer. The affinity constant K_L (L mg^{-1}) indicates the equilibrium of the adsorption process. Therefore, by plotting C_e/q_e as a function of C_e, it is possible to determine q_m from the slope of the line and K_L from its intersection with the axis.

The Langmuir isotherm has key properties that can be described by a dimensionless equilibrium parameter, known as the separation factor, which is defined as follows [6]:

$$R_L = \frac{1}{1 + K_L C_o} \quad (8)$$

Where the R_L parameter determines the type of adsorption: irreversible ($R_L = 0$), favorable ($0 < R_L < 1$), linear ($R_L = 1$) or unfavorable ($R_L > 1$); and C_0 (mg/L) is the initial concentration of mercury (II).

Text SM7. Description of the Freundlich isotherm

The Freundlich isotherm can be studied using the following equation [7]:

$$q_e = K_F C_e^{1/n} \quad (9)$$

The constant K_f is an approximate indicator of adsorption capacity, while n is a function of the strength of adsorption in the adsorption process. In the Freundlich model, values of n>1 indicate favorable adsorption conditions, whereas n<1 suggests unfavorable adsorption. A value of n=1 represents a linear adsorption process [8,9].

Text SM8. Determination of thermodynamic parameters

The changes in standard Gibbs free energy (ΔG°), standard enthalpy (ΔH°), and standard entropy (ΔS°) were calculated using the following equations [10]:

$$\ln k_c = \frac{\Delta S°}{R} - \frac{\Delta H°}{RT} \quad (10)$$

$$\Delta G° = \Delta H° - T\Delta S° \quad (11)$$

where R is the universal gas constant, T the absolute temperature (K), and Kc the apparent equilibrium constant of the adsorption process. The latter is defined as:

$$K_c = MW x 55.5 x 1000 x K_L \quad (12)$$

MW is the molecular weight of the adsorbate (Hg = 200.59 g·mol^{-1}), 55.5 represents the number of moles of pure water per liter, and K_L is the Langmuir affinity constant [11]. By plotting ln Kc against 1/T, ΔH° can be obtained from the slope of the linear regression (Fig. SM3), while ΔS° is determined from the intercept [12].

Text SM9. Relative growth index (RGI)

The relative growth index (RGI) was determined using the following equation 1 [13]:

$$RGI = \frac{SRL}{CRL} \quad (13)$$

Where SRL is the sample rootlet length, CRL the control rootlet length.

Table SM1. Composition of saline solution

Compound	Formula	Concentration (mg L^{-1})
Sodium bicarbonate	$NaHCO_3$	64.75
Calcium chloride dihydrate	$CaCl_2·2H_2O$	294.0
Magnesium sulfate heptahydrate	$MgSO_4·7H_2O$	123.25
Potassium chloride	KCl	5.75

Table SM2. Composition of the synthetic wastewater [14,15]

**Compound**	**Formula**	**Concentration (mg L^{-1})**
Magnesium sulfate heptahydrate	$MgSO_4.7H_2O$	77.4
Potassium phosphate dibasic	K_2HPO_4	10.4
Iron(III) sulfate hydrate	$Fe_2(SO_4)_3xH_2O$	209.6
Sodium sulfate	Na_2SO_4	366.9
Lead(II) nitrate	$Pb(NO_3)_2$	5.3
Citric acid	$C_6H_8O_7$	50
Ascorbic acid	$C_6H_8O_6$	30
Urea	$(NH_2)_2CO$	51
Copper(II) nitrate trihydrate	$Cu(NO_3).3H_2O$	6.1
Calcium sulfate dihydrate	$CaSO_4.2H_2O$	10.4
Zinc chloride	$ZnCl_2$	7.5

Table SM3. Interplanar spacing.

Adsorbent	2Θ (°)	Plane	d_{hkl} (Å)
RH	16.09	110	5.62
	22.06	200	4.02
	34.84	004	2.59
RHA	22	-	0.02
MCM 41	2.21	100	39.98
	3.98	110	22.20
	4.61	200	19.17
	6.02	210	14.68
	22.8	-	3.74

Table SM4. Elemental composition of adsorbents by energy-dispersive X-ray spectroscopy (EDS).

Element	RH	RHA	MCM-41

C	15.96	7.06	34.09
Al	0.21	2.73	0.25
O	50.19	46.23	42.97
Si	33.64	43.99	22.70

Table SM5. Elemental composition of adsorbents by X-ray fluorescence (XRF).

Component	RH	RHA	MCM-41
**Si**	92.4%	97.6%	99.4%
**K**	3.06%	0.49%	-
**Ca**	1.16%	0.15%	0.08%
**Cl**	1.03%	-	-
**Fe**	0.84%	0.12%	0.09%
**S**	0.42%	0.13%	-
**Mn**	0.38%	0.06%	0.03%
**Cr**	0.22%	-	-
**Ni**	0.20%	0.03%	0.03%
**Al**	0.10%	0.12%	0.16%

Table SM6. Elemental composition of MCM-41 after adsorption by X-ray fluorescence (XRF).

Element	Wt%
Si	99.99
Hg	0.01

Table SM7. Interplanar spacing post-reuse of MCM-41.

Adsorbent	2Θ (°)	Plane	d_{hkl} (Å)
MCM-41	2.15	100	40.90
	2.53	110	34.92
	3.42	200	25.83
	22.8	-	3.97

Table SM8. X-ray fluorescence analysis of industrial wastewater and river water samples.

**Component**	**Industrial wastewater**		**River water**	
	Untreated	After 24 hours of treatment	Untreated	After 24 hours of treatment
**H_2O**	99.60%	99.70%	99.9%	99.9%
**Al**	0.076%	0.079%	0.091%	0.076%
**S**	0.025%	0.025%	-	-
**Fe**	0.021%	0.020%	0.002%	0.002%
**Ni**	0.010%	0.010%	0.001%	0.001%
**Cu**	0.006%	0.006%	0.005%	0.005%
**Zn**	0.004%	0.003%	0.002%	0.003%
**Ag**	0.002%	0.002%	0.002%	0.002%
**Cd**	0.0015%	-	0.0015%	0.0014%
**Pb**	0.005%	0.004%	-	-
**Sn**	-	-	0.003%	0.004%

Table SM9. Microbiological analysis in Caquetá River. Conditions: Temperature 37°C, stirring rate 220 rpm, adsorbed dose 3.0 g L^{-1}.

**River water**	***E. Coli*** **(CFU/100 mL)**	**Total coliforms (CFU/100 mL)**	**Assessment**
Without doping Hg(II)	30	720	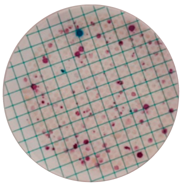
With Hg(II) 0.90 mg L^{-1}	0	0	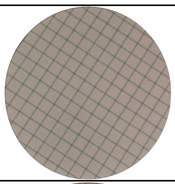
With Hg(II) and treated (MCM-41)	0	0	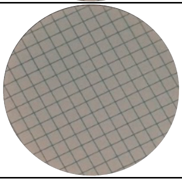
Without doping Hg(II) and treated (MCM-41)	0	0	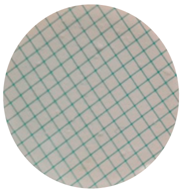

Table SM10. Classification of RGI values [13].

Inhibition of elongation	$0 < x < 0.8$
No significant effects	$0.8 < x < 1.2$
Stimulation of root elongation	$x > 1.2$, where *x* represents the calculated RGI value

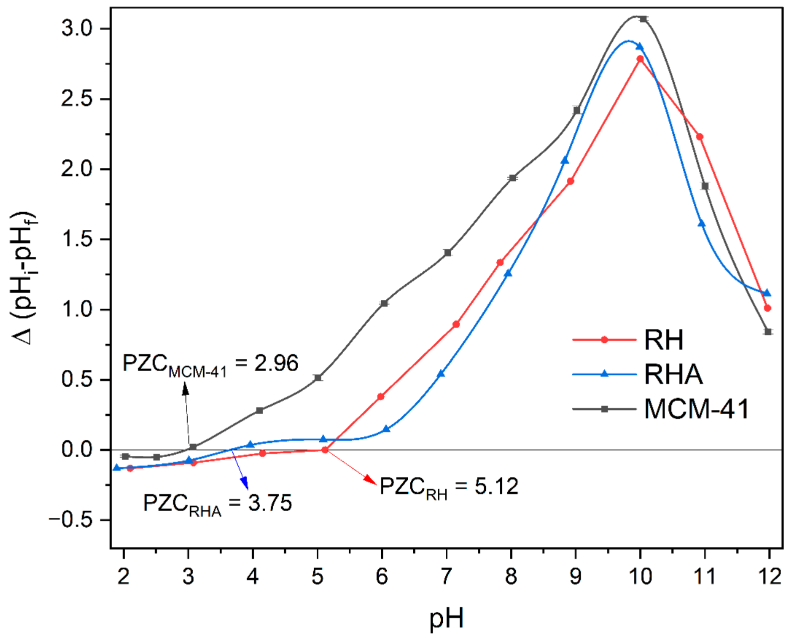

Fig SM1. Point of charge zero. Conditions: NaCl 0.1 M, adsorbent dose 1.0 g L^{-1}, stirring rate 200 rpm, temperature 25°C.

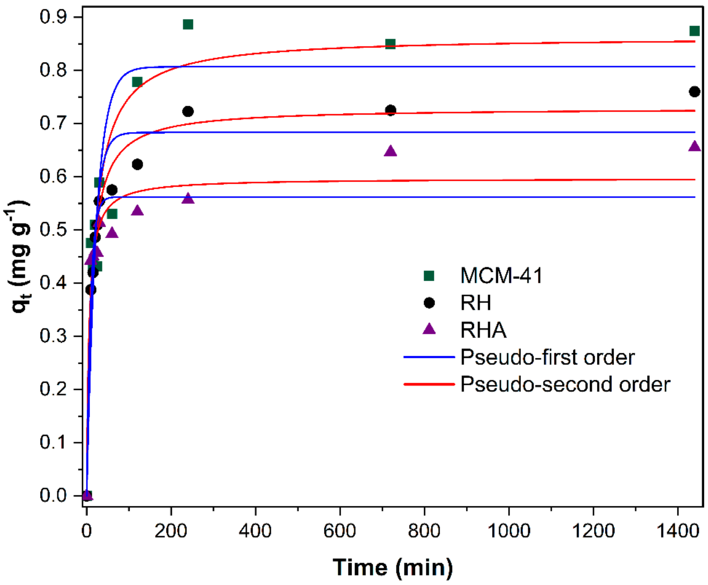

Fig. SM2. Kinetics of pseudo-first order and pseudo- second order in the removal of Hg(II) from distilled water using MCM-41, RH and RHA. Conditions: pH 6.8, initial concentration of Hg(II) 0.90 mg L^{-1}, stirring rate 270 rpm, adsorbent dose 0.5 g L^{-1}, temperature 25 °C.

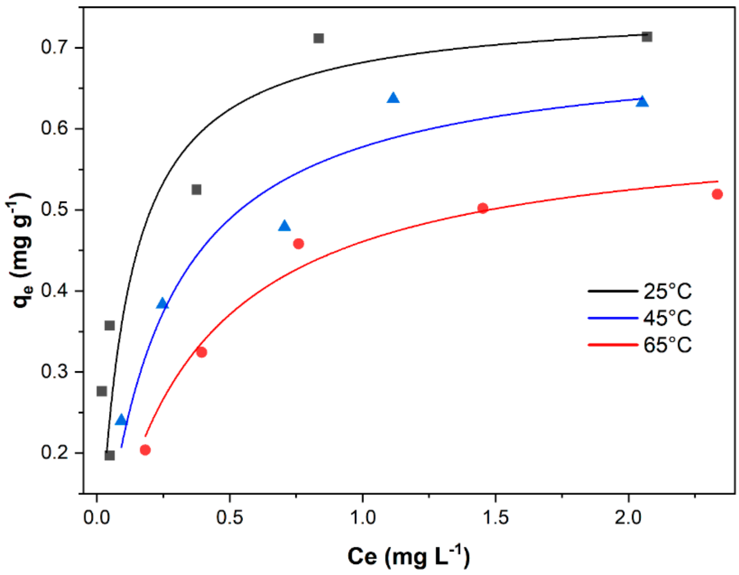

Fig. SM3. Adsorption capacity at different temperatures. Conditions: pH 6.8, stirring rate 270 rpm, time 12 h, initial concentration of Hg(II) 0.90 mg L^{-1} and adsorbent dose 3.0 g L^{-1}.

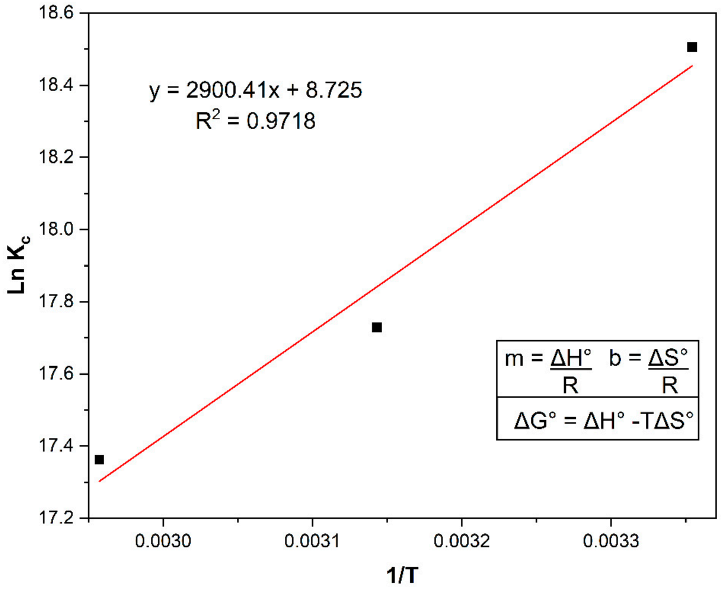

Fig SM4. ln K_c vs. 1/T to obtain the thermodynamic parameters in the Hg(II) adsorption. Conditions: Hg(II) concentration 0.90 mg L^{-1}, MCM-41 dose 3.0 g L^{-1}, pH 6.8, time 12 h, temperature 25-65 °C.

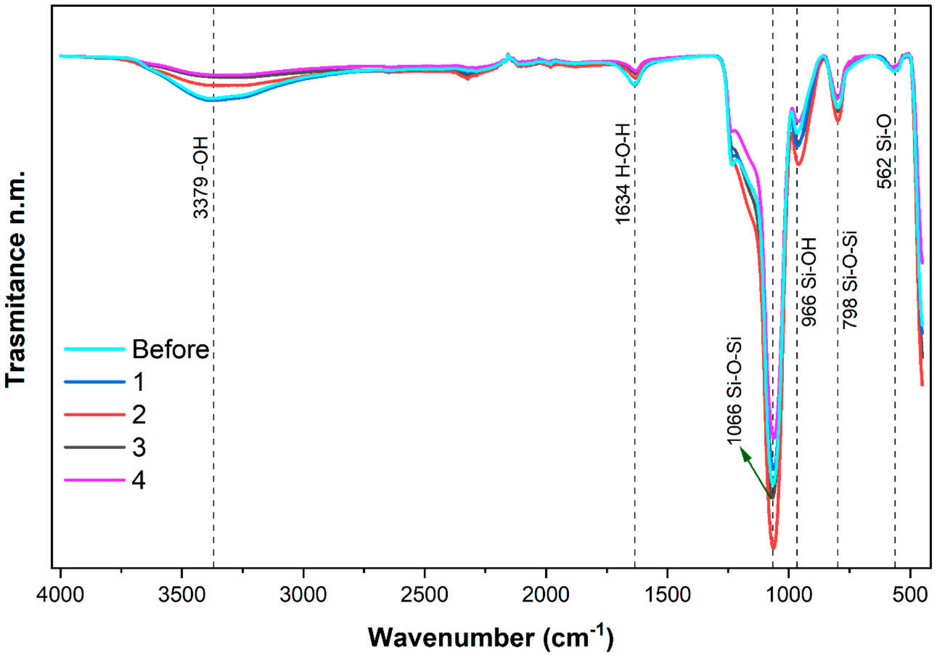

Fig SM5. FTIR spectra of MCM-41 before and after four adsorption cycles of Hg(II). Conditions: pH 6.8, stirring rate 270 rpm, time 24 h, adsorbent dose 3.0 g L^{-1}, initial concentration of Hg(II) 0.90 mg L^{-1}.

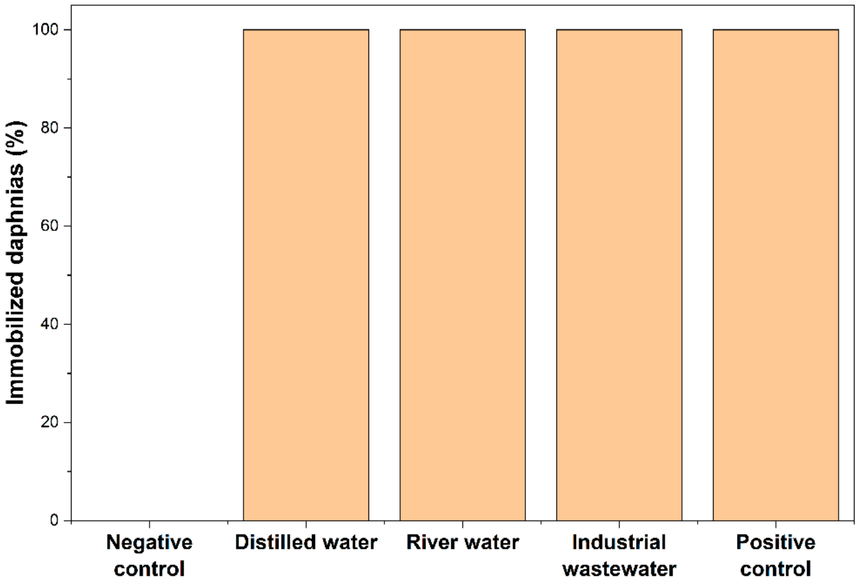

Fig. SM6. Immobility of *Daphnia magna* in three water matrices. Conditions: pH 6.5, temperature 22 °C, time 24 h.

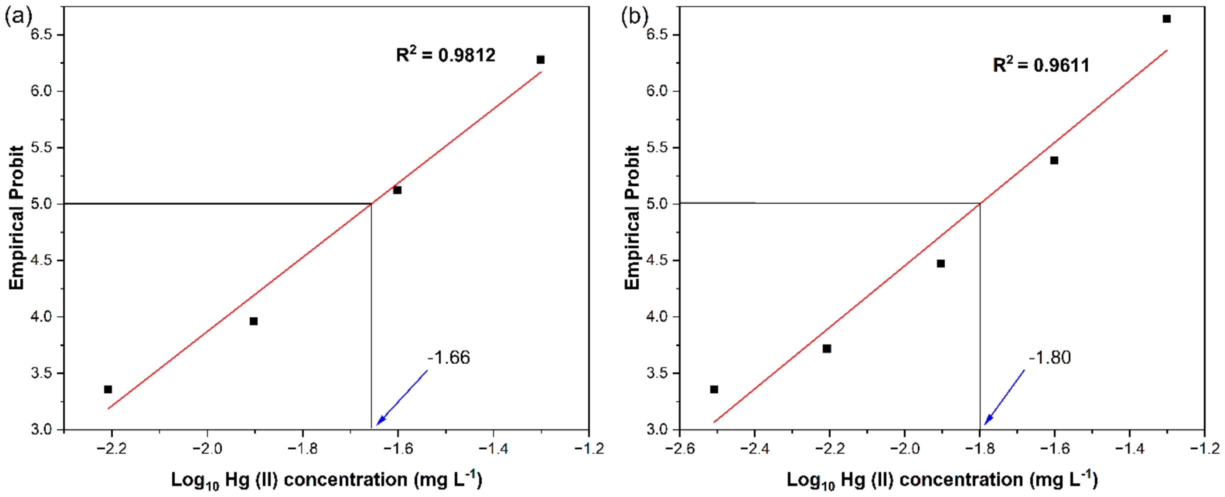

Fig SM7. Probits. (a) 24 h, (b) 48 h. Conditions: *Daphnia magna* maintained at 22 °C in distilled water supplemented with saline solution (Table SM1) using Hg(II) concentrations ranging from 0.0031 to 0.075 mg L^{-1}.

References

1. Yokoi, T.; Nakamura, J.; Ohtsuki, C. 7 - Incorporation Behavior and Biomedical Applications of Inorganic-Layered Compounds. In Bioceramics; Osaka, A., Narayan, R., Eds.; Elsevier Series in Advanced Ceramic Materials; Elsevier: Netherlands, 2021; pp. 139–158.
2. Jendrzejewska, I.; Musioł, R.; Goryczka, T.; Pietrasik, E.; Klimontko, J.; Jampilek, J. The Usefulness of X-Ray Diffraction and Thermal Analysis to Study Dietary Supplements Containing Iron. Molecules **2021**, *27*, 197.
3. Rahim, A.A.A.; Miskolczi, N.; Ateş, F. Removal of Insecticide Carbofuran from Aqueous Solutions by Activated Carbon/Polyaniline Composites. Desalination Water Treat. **2025**, *323*, 101381.
4. Chu, K.H.; Bollinger, J.-C.; Kierczak, J. Pseudo-First-Order Kinetics in Environmental Adsorption: Why Are There Two Distinct Equations? Environmental Surfaces and Interfaces **2025**, *3*, 191–195.
5. Sun, Z.; Tian, C.; Yang, T.; Fu, J.; Xu, H.; Wang, Y.; Lin, Z. A MOF-Based Trap with Strong Affinity toward Low-Concentration Heavy Metal Ions. Sep. Purif. Technol. **2022**, *301*, 121946.
6. Imla Syafiqah, M.S.; Yussof, H.W.; Kinetics, Isotherms, and Thermodynamic Studies on the Adsorption of Mercury (II) Ion from Aqueous Solution Using Modified Palm Oil Fuel Ash. Mater. Today Proc. **2018**, *5*, 21690–21697.
7. Nasir, M.Z.M.; Zaini, M.A.A.; Yunus, M.A.C. Adsorption Profiles of Rhodamine B and Reactive Orange 16 onto Pharmaceutical-Based Activated Charcoals. Desalination Water Treat. **2018**, *132*, 340–349.
8. Chiban, M.; Soudani, A.; Sinan, F.; Persin, M.; Single, Binary and Multi-Component Adsorption of Some Anions and Heavy Metals on Environmentally Friendly Carpobrotus Edulis Plant. Colloids Surf. B Biointerfaces **2011**, *82*, 267–276.
9. Dada, A.O.; Ojediran, J.O.; Olalekan, A.P. Sorption of Pb2+ from Aqueous Solution unto Modified Rice Husk: Isotherms Studies. Advances in Physical Chemistry **2013**, *2013*, 842425.
10. Medici, F.; Patterer, M.S.; Peluso, M.A.; Sambeth, J.E. Lead Adsorption from Aqueous Solution Using Manganese Oxides Recovered from Spent Alkaline Batteries. Journal of Solid Waste Technology and Management **2020**, *46*, 206–212.
11. Paredes-Laverde, M.; Silva-Agredo, J.; Torres-Palma, R.A. Removal of Norfloxacin in Deionized, Municipal Water and Urine Using Rice (Oryza Sativa) and Coffee (Coffea Arabica) Husk Wastes as Natural Adsorbents. J. Environ. Manage. **2018**, *213*, 98–108.
12. Cantu, Y.; Remes, A.; Reyna, A.; Martinez, D.; Villarreal, J.; Ramos, H.; Trevino, S.; Tamez, C.; Martinez, A.; Eubanks, T.; et al. Thermodynamics, Kinetics, and Activation Energy Studies of the Sorption of Chromium(III) and Chromium(VI) to a Mn3O4 Nanomaterial. Chemical Engineering Journal **2014**, *254*, 374–383.

13. De Almeida, M.; Machado, M.R.; Costa, G.G.; de Oliveira, G.A.R.; Nunes, H.F.; Maciel Costa Veloso, D.F.; Ishizawa, T.A.; Pereira, J.; Ferreira de Oliveira, T. Influence of Different Concentrations of Plasticizer Diethyl Phthalate (DEP) on Toxicity of Lactuca Sativa Seeds, Artemia Salina and Zebrafish. Heliyon **2023**, *9*, e18855.
14. Lastre-Acosta, A.M.; Palharim, P.H.; Barbosa, I.M.; Mierzwa, J.C.; Silva Costa Teixeira, A.C. Removal of Sulfadiazine from Simulated Industrial Wastewater by a Membrane Bioreactor and Ozonation. J. Environ. Manage. **2020**, *271*, 111040.
15. Patel, P.K.; Pandey, L.M.; Uppaluri, R.V.S. Adsorptive Removal of Zn, Fe, and Pb from Zn Dominant Simulated Industrial Wastewater Solution Using Polyvinyl Alcohol Grafted Chitosan Variant Resins. Chemical Engineering Journal **2023**, *459*, 141563.
